# Functional Equivalency Inferred from “Authoritative Sources” in Networks of Homologous Proteins

**DOI:** 10.1371/journal.pone.0005898

**Published:** 2009-06-12

**Authors:** Shreedhar Natarajan, Eric Jakobsson

**Affiliations:** 1 Biophysics and Computational Biology, University of Illinois, Urbana-Champaign, Illinois, United States of America; 2 National Center for Supercomputing Applications, University of Illinois, Urbana-Champaign, Illinois, United States of America; 3 Department of Molecular and Integrative Physiology, University of Illinois, Urbana-Champaign, Illinois, United States of America; Indiana University, United States of America

## Abstract

A one-on-one mapping of protein functionality across different species is a critical component of comparative analysis. This paper presents a heuristic algorithm for discovering the Most Likely Functional Counterparts (MoLFunCs) of a protein, based on simple concepts from network theory. A key feature of our algorithm is utilization of the user's knowledge to assign high confidence to selected functional identification. We show use of the algorithm to retrieve functional equivalents for 7 membrane proteins, from an exploration of almost 40 genomes form multiple online resources. We verify the functional equivalency of our dataset through a series of tests that include sequence, structure and function comparisons. Comparison is made to the OMA methodology, which also identifies one-on-one mapping between proteins from different species. Based on that comparison, we believe that incorporation of user's knowledge as a key aspect of the technique adds value to purely statistical formal methods.

## Introduction

The current spate of genome sequencing projects [1] has resulted in large amounts of sequence information from all kingdoms of life. Experimental techniques to characterize and annotate these sequences have not yet kept pace with the generation of data, and it is not foreseeable that they ever will, because sequencing is inherently faster than all present or foreseeable methods of experimental functional determination. Therefore, comparative genomic analysis is being increasingly employed for functional annotation. The basis of most comparative techniques is the notion of homology or common evolutionary origin of the gene/protein sets being investigated. The multiplicity of evolutionary scenarios necessitates a more fine-grained description of homology in terms of orthologs, in-paralogs and out-paralogs [2]. Orthologs are genes from different species that have a common ancestor. Traditionally, orthologous genes from different species were thought of as having similar functions. However, gene duplication can result in functional divergence within a species and give rise to paralogs. In-paralogs and out-paralogs are defined based on the relative order of duplication and speciation events. Depending on the degree of divergence, paralogs can retain a significant portion of the sequence features of the original gene. Since duplication of a gene can still satisfy the constraint of common ancestor with genes from other species, multiple pairs of orthologous genes in two species can have arisen from a single ancestor prior to the duplication.

Our explorations were motivated by a desire to predict protein interaction networks using the evolutionary correlation method [3]. This method is based on the premise that proteins that interact would have correlated substitution patterns across species. Application of the evolutionary correlation method requires a protocol to identify corresponding proteins for the comparison. It is desirable that the full repertoire of functional capabilities of each protein - both in terms of its physiological roles, as well as the mechanisms of regulation - be as similar as possible across the species set considered. Imposing this constraint will also likely ensure that the protein pair from each species interacts with each other. In the absence of prior knowledge on the multiplicity of pairings between the two protein sets, it is necessary that the protein representatives be unique for each species. In our work, we refer to such a sample as the **most likely functional counterpart (MoLFunC)** of each other.

A pair of “MoLFunCs” is similar to a pair of orthologous proteins, but the concept is slightly different. The strict definition of orthology is in terms of descent. The root definition of orthology is in terms of genes, and the application to proteins is derived from the application to genes. The definition of “MoLFunC” is specific to proteins, and implies an attribution of a common function. Note that in the definition of MoLFunCs, different splice variants of orthologous genes may not be MoLFunCs of each other.

The most common tool used for sequence similarity is BLAST – Basic Local Alignment Search Tool [4]. It often happens that the result of bi-directional BLAST searches between two genomes is asymmetric. If protein *PA* in species *A* picks up protein *PB* in species *B* as the most significant hit, it is not necessary that protein *PB* pick up *PA* in species *A*. This asymmetry could be used to restrict the orthology requirement to symmetric best-best hits [5]. By comparing all genomes to all other genomes, the best-best hits could be daisy-chained until they ended in a closed loop. However, this criterion may in some cases be unduly restrictive and one may begin with a large number of species and end up with very few species having the “true” ortholog of the protein being investigated. The problem is in the requirement that symmetrical best hits are required for all organism pairs, so that any one failure of this requirement breaks the daisy chain.

There have been several efforts to systematically catalog orthologous genes/proteins from several species [6]–[11]. Most techniques have employed whole genome comparisons with clustering algorithms and/or clique finding, to group similar genes based on their similarity scores. Large-scale genome comparisons are highly resource-intensive and therefore databases containing orthologous groups are updated slowly. Moreover, many of the orthologous groups have multiple representatives from each species, possibly due to the allowance of asymmetry in reciprocal BLAST hits. This scenario is further complicated in higher organisms where a significant portion of functional diversity is also achieved through alternative splicing.

Although all previous efforts have used rigorous criteria in defining orthologs, it was not intuitively clear as to how we could extract MoLFunCs from these datasets. Several techniques have been proposed that attempt to filter out paralogs; but it was difficult to both define and ascertain the level of consistency that these filtering techniques would have with the original clustering based methods. More recently, efforts were made to identify ortholog groups with single representatives from each species [11]. However, in spite of the overall broad coverage of species, a preliminary examination of some of the proteins revealed a sample that did not include species that we predicted would contain the proteins. In such cases and in the case of most existing resources, it was not apparent as to how user confidence or expectations could be included in the MoLFunC identification process seamlessly, consistently and efficiently with the existing dataset. A related problem is that of adding newly identified or curated sequence sets for an existing species, or a new species set, and reconstructing the MoLFunCs to reflect the updated knowledge.

Large-scale ortholog identification efforts are very useful in revealing global statistical patterns of protein evolution, and are especially valuable in guiding genome-wide experimental efforts and analysis. However, focused biophysical explorations that seek to study thoroughly a few proteins, would benefit from a flexible, yet rigorous platform to identify MoLFunCs for comparative analysis of those proteins and related ones. As a first step toward building a tool that can be guided by user knowledge, we developed a method that relies on the simplest of such cases, viz., high-confidence functional annotation of the proteins being investigated. We have attempted to retain the rigorous techniques espoused by former approaches, while also identifying a common theme that can be consistently applied at every step of our algorithm. Since experimental validation of the functional equivalency of each protein in the dataset is a difficult task [12], we provide verification in the form of necessary, if not sufficient, conditions that the MoLFunC set should satisfy.

Our initial test bed was Kv1.2, a voltage gated potassium channel from rat, whose 3D structure was recently solved by Mackinnon et.al [13]. Kv1.2 was chosen due to the wealth of information available for voltage gated K+ channels. Voltage gated potassium channels are a diverse family of ion channels that allow selective permeation of K+ ions at specific transmembrane voltages [14]. There are a large number of genes encoding potassium channels in eukaryotes, in addition to their wide distribution in microbial species. In excitable cells such as neurons, potassium channels are an important contributor to the resting membrane potentials and action potentials. They are also important pharmaceutically and are the targets of several toxins that bind specific regions of these channels with high affinity. Kv1.2 is a Shaker-like potassium channel, named after the initial identification of the Shaker gene in the fruit fly [15]. The Kv1.2 channel is a homo-tetramer, with each monomer consisting of 6 transmembrane helices, S1–S6. S1–S4 serves to sense voltage changes across the membrane, while S5–S6 form the pore region that facilitates K+ permeation. The channel can open or close depending on the transmembrane potential, and the part of the pore that widens on opening is the portion of S6 near the intracellular side. It is known that potassium channel function is modulated by auxiliary Beta subunits that are homologous to the oxidoreductase family of proteins [16]. The 3D structure of Kv1.2 includes the Beta2 subunit from rat [13]. Since this channel has S1, S4, S5–S6 and the beta subunit, it provides the structural counterpart of many aspects of the full functionality of the channel protein and, together with the large amount of functional data, serves as an indicator for the reliability of functional annotation. In addition, we tested our technique on 6 other membrane proteins (Table 2), for which topological analysis is available in literature and for which specific residues/motifs have been identified as important for function.

## Methods

### Theory

An inspiration for our approach was drawn from an analogy between the network of protein homologies and network structures in other domains of knowledge such as social networks and the World Wide Web (WWW). Most of these networks have directed edges between nodes. In the case of the WWW, the direction indicates a link from one website to another, while in social networks, the direction could indicate flow of rumor or gossip. In the case of the protein homology network, the direction indicates the direction of BLAST search, and the edges can carry weights proportional to the score, e-value, percent identity or rank. Early research on the management of information on the WWW [17] sought to exploit the link structure of the Internet to improve search engine performance and accuracy. One hallmark of these efforts was the definition of authorities and hubs. Authorities are websites that can have a high degree of incoming links, while hubs have a high degree of outgoing links, primarily to authorities. Analysis of the equilibrium between different types of nodes helped fashion a search algorithm that could identify relevant websites to user queries. The twin ideas of “authority” and “incoming links” served as appropriate metaphors to map into our own problem domain.

Since in our case, the function of the protein is well known and enjoys a high level of confidence, the protein is *authoritatively annotated*. We refer to the species containing the most authoritatively annotated protein as the *authority species* and the protein itself as the *authority*. An inbound link to protein B from protein A in the protein homology network refers to protein B being the best hit in a homology search with protein A as the query against the genome of protein B. The concepts of *authority* and *incoming links* can thus be applied to whole genome searches by stipulating that any protein which is functionally equivalent to an authority should necessarily pick up the authority as the best hit when searching against the genome of the authority species.

We now introduce an analogy to social networks to extend our strategy. *Authoritative annotation* can be viewed as a *factoid* or a *rumor or a piece of gossip* (although in this case we believe the gossip to be true). The problem of identifying MoLFunCs can be viewed as diffusion of gossip (annotation information) among other proteins in all species. Gossip starts from a single source, presumed to be an authority on the subject of the gossip. The source may share the information with many others (analogous to the authority picking best hits form another genome): however the gossip spreads further only by those receivers who believe in the gossip (analogous to picking up the authority as the best hit). This *belief* is expressed as the incoming link to an authority from another node. Each of these nodes thus becomes the *authority seed* to identify the next generation of gossip believers. Note that we retain the flavor of consensus agreements included in previous approaches by demanding that every protein other than the original authority needs to have a degree of *authoritativeness* (but a lower degree than the original authority). The high degree of confidence in the functional annotation of the protein used to start the algorithm is treated as “prior knowledge supplied by the user”. Since the method depends on the starting point, the identification of the authority protein is the “user input”. We now outline our specific methodology that was based on the above theoretical considerations.

### Algorithm

**Figure 1:**
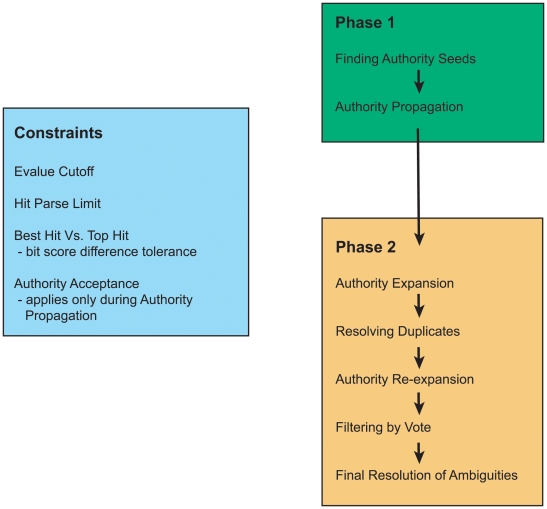
Overall workflow for finding MoLFunCs.

Since the reciprocal homology search technique is a necessary precursor to most of the ortholog prediction methods, we based our strategy on similar grounds but with slight modifications. As the reciprocal best hits technique could result in a very low sample size, we bias the requirement of “best hit” in an appropriate direction and also relax the definition of “best hit” vis-à-vis the “top hit”. This allows a larger sample to be accrued, but with several species contributing more than one MoLFunC. We then exhaustively and iteratively refine the putative list of MoLFunCs using profile analysis tools like HMMER [18] to arrive at a unique protein for each species. Figure 1 details the overall workflow.

### Phase I - exhaustive identification of MoLFunCs

This phase involves repeatedly running BLAST searches and arriving at a list of possible MoLFunCs from all species. For convenience, we refer to a BLAST search initiated by the authority or an authority seed as the forward-BLAST, the hits obtained from the forward-BLAST as the reverse-query, and the BLAST of the reverse query with the genome of the authority or an authority seed, as the reverse BLAST.

#### Establishing new authority seeds

We start by doing a BLAST search on every relevant genome, both complete and incomplete (possible errors from incomplete genomes will be filtered out at a later stage of the workflow), using the authority protein as the query. For each BLAST result, we go through each hit in ascending order of e-value and perform a *reverse* BLAST search of the hit against the query genome. The first hit sequence that retrieves the original authority protein in the reverse blast as its best hit is designated as a putative MoLFunC. Thus we bias the bi-directional BLAST hits procedure in the direction of the authority. At the end of the first iteration, we have a list of proteins from many species, each of which pick up the authority as the best hit.

#### Authority Propagation

Each of the sequences obtained in the first iteration is used as an *authority seed* for a further iteration of BLAST searches against all the genomes. After the first iteration, every reverse-query obtained by an authority seed is also searched against the original authority genome with the constraint that it should pick up the original authority as the best hit. Thus every MoLFunc at the end of the code execution would have picked up the authority as the best hit. We refer to this as *authority acceptance*. If there is complete consensus, the second iteration should still result in the same set of putative MoLFunCs from other species as was obtained in the first iteration. However, we often find that subsequent iterations pick up new sequences from other genomes that are in turn, seeds for further iterations. Iterations are repeated until no new sequences can be found. At this point it may be that there has been accepted more than one protein per genome.

### Heuristics

The computations above were fairly compute-intensive, due to the large number of BLAST searches. Since we examine each hit in the forward BLAST until we find one that picks the query as best hit in the reverse BLAST, we could end up parsing a large number of hits making the algorithm very time-consuming. Therefore we decided to introduce some heuristics to aid in convergence, as described below.

#### Threshold

For all BLAST searches, we only considered hits that had an **e-value of less than 1e-10**. While processing the results of a forward BLAST, we process only up to **10 hits**. Both these cutoffs limit the accrual of false positives and help reduce the time taken to run the algorithm.

#### Best hit vs. Top hit

We allowed for the possibility that the top hit is not necessarily the best hit. We allowed for a margin of error, i.e., variability, to be included in the form of a bit difference threshold. One possible source of variability is choice of the specific substitution matrix that is used for the BLAST. And further, as databases are continually curated and updated, the exact sequences returned may vary. Thus it is possible that the second hit is almost as good as the top hit except for a difference of a few bits (based on the Bit score reported) while being as significant as the top hit. Although the actual sequences of the two hits may differ from each other in more than a couple of positions, from the perspective of the query, the two sequences are almost alike. Thus, allowing for a margin of error, it would be acceptable to choose a hit with lower bit similarity over the top hit. We constrain the bit difference to be no more than 10 bits, which was arrived at by trial-and-error.

We use this constraint in situations where a hit representing a new putative MoLFunC is only slightly better than a hit representing a previously identified authority or putative MoLFunC. In these cases, we discard the new hit. This can occur in two places in the workflow; in the reverse BLAST searchers against the authority or authority seeds, and in the forward BLAST, to search for hits that are below the top hit, but might already be obtained as a putative MoLFunC in an earlier iteration or from the result of starting from another authority seed. The reverse BLAST constraint also respects the limit of 10 hits.

#### End of Phase I of the Workflow

**Figure 2:**
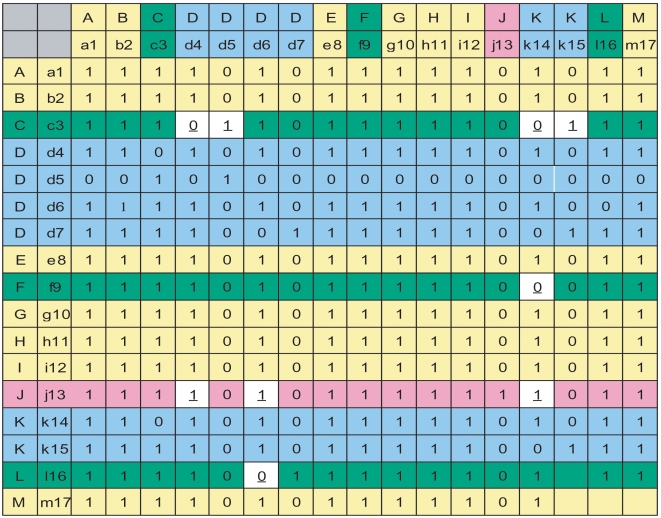
A sample MoLFunC Matrix at the end of Phase 1 in the Workflow (Figure 1). The first row and column contains species symbols and the second row and column contains corresponding proteins. Each matrix element is binary; *“1”* indicates the column protein picked the row protein as best hit in the reverse BLAST, *“0”* indicates that there is no orthology relation discovered between the row and column proteins at this stage of the workflow. Species are color-coded to fit in four categories—pink is the species containing the original authority protein (Figure 1); yellow are species for which one MoLFunc was unambiguously established, by complete agreement with the origin authority species; green are species for which one MoLFunC was determined, but there is not agreement with the original authority species, and blue are species for which multiple proteins survived the Phase 1 process as putative MoLFunCs. Matrix elements that define the ambiguous species by virtue of difference from the original authority species are colored in white and their values are underlined and in bold. For the green and blue species, final MoLFunC determination occurs in Phase 2 of the workflow.

The end result of this analysis is a binary matrix with identical row and column indices consisting of all possible MoLFunCs (Figure 2). Each cell is a “1” or a “0” indicating that the column sequence was found as a putative MoLFunC when starting the BLAST search from the row sequence (i.e. authority or an authority seed). We refer to this matrix as the *MoLFunC Matrix*. There can be more than one protein per species and we need to filter out the true MoLFunCs to arrive at one protein from each species.

### Phase II - Refinement for unique MoLFunCs

In the refinement step, we continue to use the concept of authority in constructing profiles and searching relevant genomes using HMMER [18]. The procedure builds on the idea of authority by first constructing an *authoritative core* (to be defined and described below) from the MoLFunC matrix and then using this core to resolve uncertainties among the non-core sequences. Uncertainties are categorized into two types – *duplicates* and *ambiguous* sequences. Any species having two or more representative sequences is termed *duplicate*. Each row vector in the MoLFunC matrix gives an indication (described above) of how authoritative the row sequence is. In order to determine the core set, we compare the row vectors of all non-duplicate species with that of the authority. The species and the corresponding sequences that match the authority are included in the core set. The argument here is that while calculating MoLFunCs, the authoritativeness was checked with respect to the entire genome, whereas in the refinement step, the authoritativeness is checked with respect to all the MoLFunCs. So the matching of the row vectors suggests that the sequence with that vector is as authoritative as the initial authority, with respect to the rest of the MoLFunCs. Any species with unique representatives, but having a different bit vector than the authority (the initial probe), is termed *ambiguous*. The core determination is a form of *authority expansion*, as the information content of the original authority is augmented with newer authoritative sequences, while the resolution of ambiguities and duplicates is a form of *authority driven verification*.

#### Authority Expansion

We align the core set sequences using MUSCLE [19] and, using HMMER (*hmmbuild* command), build the core profile. The profile is in the form of a numerical matrix that seeks to capture the average information content in the sequences. We use this profile to search species that contain ambiguous or duplicate sequences. If the core set consists of only the original authority, then there is no need to construct an alignment or a profile.

#### Duplicate resolution

For each species with multiple MoLFunCs, we use HMMER (*hmmsearch* command) to search the genome of the species with the core profile. We use a bit difference threshold of 10 bits to allow including hits that might be already in the MoLFunC matrix but not the very top hit of the HMMER search result. In case we find a hit that is not already included and no other MoLFunC sequence is within 10 bits, we exclude the species from the MoLFunC matrix. If the core set consists of only the original authority, then the protein that was picked up by the authority in the first step is chosen. If none of the proteins were chosen in the first round, then the species is removed from the MoLFunC matrix.

#### Authority Re-expansion

After removing the appropriate duplicates from the initial MoLFunC matrix, we now have a *reduced MoLFunC matrix* with unique proteins from each species. We repeat the core calculation process and construct a profile from the new core set. Again, if the core consists of only one sequence, there is no need to align or construct a profile; the next two steps are skipped and the reduced MoLFunC matrix is treated as the final MoLFunC matrix.

#### Filtering by Vote

**Figure 3:**
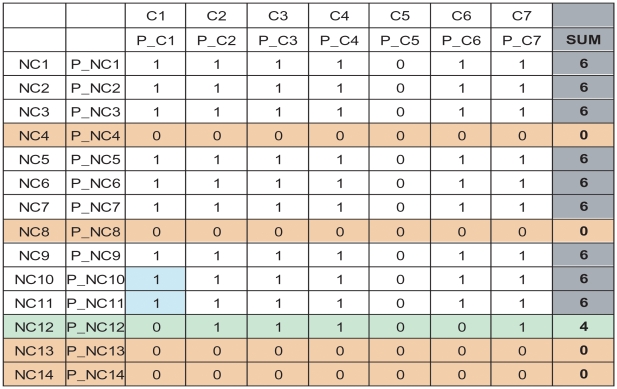
Subset sample of the reduced MoLFunC matrix after duplicate resolution and Authority Re-Expansion (see workflow in Figure 2). This figure represents filtering by vote and final resolution of ambiguities by HMMSearch. The first row and column are species names and the second row and column represent protein ids. The column headings represent fully resolved “core” MoLFuncs; i.e. yellow species from Figure 3 plus those green species whose ambiguity was removed and the blue species whose multiple candidates were successfully eliminated by the first three steps in Phase 2 of the workflow. The row indices indicate species and proteins that are questionable because of still-unresolved disagreements with the authority species. The white matrix elements indicate rows that agree with the core MoLFuncs, and are therefore accepted as MoLFuncs. The blue cells indicate that there was residual ambiguity that was resolved by being best hit from HMMSearch. All the species/proteins whose row sum was 6 (gray cells) were accepted into the final set. Beige cells indicate substantial disagreement with the core MoLFuncs; these species/proteins are discarded and do not appear in the final set. The species with the green cells had intermediate level of agreement with the core MoLFunCs, and the ambiguities were not picked as best hit by HMMSearch; this species and protein were thus discarded. The column with all entries as zeros is the original authority; the cells are zero because we never do a reverse BLAST starting from the original authority as the query.

In the next stage of the workflow, we filter out some of the ambiguities, before performing a costly profile search. For this purpose, we extract a subset of the *reduced MoLFunC matrix*, with the rows corresponding to the most recent non-core sequences, and the columns corresponding to the most recent core set (see Figure 3) The row components found the column components as one of the top ten hits in that species. The value in each matrix element is 1 if the column found the row as the best hit, and 0 otherwise. The **sum of each row** is interpreted as a measure of how authoritative all the non-core species are with respect to the new core set. For any row whose sum is less than half the maximum; i.e. less than half the number of columns, the sequence corresponding to that row is removed from any further analysis. Figure 3 shows an example of this filtering.

#### Ambiguous sequence resolution

The rest of the ambiguous species are resolved in a similar fashion as the duplicates, by searching the species genome with the new core profile and extracting the relevant hit, if any. Again, if we find a best hit that is not included and no other MoLFunc sequence is within 10 bits, we exclude the species from our analysis.

#### End of Phase II

The final result is a MoLFunC matrix with one representative per species. Note that the refinement procedure allows the core authority to pick the “best hit” from the HMMER result as opposed to the core being selected by the “best hit” (which was used in Phase I for authority propagation). We can do this because we are now pruning existing MoLFunCs rather than discovering new ones and there is only one profile to search against, rather than an entire genome.

### Summary of Workflow

A simple analogy can aid in understanding the overall process. Our goal is to construct a council of authoritative members (proteins) who best represent their respective constituencies (proteomes). It is important that all council members are able to work with each other as well as with the chief authority that initiates the selection process. The very first step is for the chief authority to identify candidates in each proteome (forward BLAST) and selecting the representative who fully acknowledges the chief's authority (chooses the chief authority as the best hit in a reverse BLAST). Once the representatives have been identified, the next step is to acknowledge each other's authority and build respect and cooperation. This is carried out in the authority expansion step, in which new representatives may be nominated from some of the proteomes as being more compatible with some of the existing members. This process is repeated until we have a putative council consisting of disagreements between some of the members (cells that have a 0 in the MoLFunC matrix) as well as multiple representatives from some proteomes.

The refinement step builds a set of core representatives by comparing the authority profiles of all members (except members from proteomes with multiple representatives) with the chief authority. This builds a circle of trust and their consensus view is represented by a profile (using HMMER). Those members that are outside this circle, but uniquely represent their proteome are called the non-core proteins. The consensus profile is used to search the proteomes with multiple entries to decide on which representative to retain (best hit in HMMER search). If none prove to be trustworthy, that proteome is excluded from representation. The filtering by vote strategy checks if most members of the core individually accepted the authority of the non-core proteins. If the degree of acceptance is very low, they are culled from the list. The remaining non-core proteins are then confirmed using the consensus profile search against the respective proteome. One problem that can arise in the refinement step is that the core may end up consisting of only the original authority. In this case, simply choosing the protein that featured in the very first search from the authority solves the multiple-representative problem. The rest of the disagreements between members are left as an unresolved issue at this time. In our discussion section, we have outlined a possible strategy to further refine our dataset.

### Implementation

The Kv1.2 sequence from Rat was chosen as the authority for the Kv1.2 calculations because the 3D structure was determined for the Rat sequence. For the rest of the proteins, we identified authorities on the basis of their record in the TCDB [20]. The sequences extracted from these records were then compared (using BLAST) with the RefSeq [21] database for the corresponding species and the top hit chosen as the authority sequence that was fed into the algorithm.

We included all possible metazoan genomes from the Refseq [21] database at NCBI (downloaded on 09/07/07) and the ENSEMBL [22] database at EBI (Release 46). We decided to search through both sets of databases, as we were unsure of the overlap between the two. Table 1 shows the initial species set considered from both databases. Initially we determined two sets of MoLFunCs, one from each database. We then merged the sets of MoLFunCs in the following fashion:

**Table 1:** List of species covered using NCBI and ENSEMBL.

SPECIES	NCBI	ENSEMBL
Aedes aegypti		X
Anopheles gambiae	X	X
Apis mellifera	X	
Bos taurus	X	X
Caenorhabditis elegans	X	X
Canis familiaris	X	X
Cavia porcellus	X	X
Ciona intestinalis	X	X
Ciona savignyi	X	X
Danio rerio	X	X
Dasypus novemcinctus	X	X
Drosophila melanogaster	X	X
Drosophila pseudoobscura	X	
Echinops telfairi	X	X
Equus caballus	X	
Erinaceus europaeus	X	X
Gallus gallus	X	X
Gasterosteus aculeatus	X	X
Homo sapiens	X	X
Loxodonta africana	X	X
Macaca mulatta	X	X
Monodelphis domestica	X	X
Mus musculus	X	X
Myotis lucifugus		X
Ornithorhynchus anatinus	X	X
Oryctolagus cuniculus	X	X
Oryzias latipes	X	X
Otolemur garnettii		X
Pan troglodytes	X	X
Rattus norvegicus	X	X
Sorex araneus		X
Spermophilus tridecenlineatus		X
Strongylocentrotus purpuratus	X	
Takifugu rubripes	X	X
Tetraodon nigroviridis	X	X
Tribolium castaneum	X	
Tupaia belangeri	X	X
Xenopus laevis	X	
Xenopus tropicalis	X	X
Sus scrofa	X	

**Table 2:** List of proteins studied and the species with the authoritative annotation.

Protein	Authority Species
Kv1.2 (NP_037102.1)	Rat
HCN1 (NP_034538.1)	Mouse
Trpv1 (NP_114188.1)	Rat
Glur (NP_113796.1)	Rat
Cng1 (NP_000078.2)	Human
Kir7.1 (NP_002233.1)	Human
Gamma6 (NP_542425.1)	Rat

Since we started with the Refseq sequences, we decided to take the Refseq entry if the ENSEMBL entry picked it up as the best hit. In case there was ambiguity in the BLAST hit of ENSEMBL sequence vs. the Refseq database for that species, we did a pairwise global alignment of the original probe with each of the NCBI and ENSEMBL sequences and calculated the distance matrix for the alignment using CLUSTALW. The sequence that was closer to the original probe was taken as the MoLFunC. For example, in the Kv1.2 calculation, the only ambiguity was in the case of Drosophila; the ENSEMBL version was finally chosen, as it was closer to the original probe.

### Topological Congruence

We used TMHMM v2.0 [23] to determine the transmembrane topology of the MoLFunCs. One weakness of TMHMM that applies to voltage-gated channels is that it may not predict the pore helix and/or the S4 region in some proteins. We observed that the reason for this is that in many voltage-gated channels, these regions fall below the threshold for TM probability. We wrote a Perl script to reparse the raw TMHMM output using heuristics to eliminate local (less than 7 residues from one peak to start of next and less than 3 residues from start of current TM to its peak) maxima. This permitted us to use a threshold of 0.2 for TM probability for the voltage-gated channels (compared to 0.4 standard for TMHMM). For the rest of the proteins, we used the standard TMHMM prediction. Each of the MoLFunCs was aligned globally to the corresponding authority protein using MUSCLE [19] and the residue mapping between the two sequences was extracted from the alignment. In order to examine topological congruence, we constructed a dot matrix from the residue mapping, using MATLAB. We then parsed out the TM profiles for the authority protein and the aligned MoLFunC, and visually superimposed them on the plot so we could see the alignment in the context of the topologies.

### Conserved Motif Analysis

A multiple sequence alignment was carried out using MUSCLE [19] and the conservation patterns of the relevant motifs were inferred from the alignment. This step was carried out after the check for topological congruence to avoid including incorrect MoLFunCs that might perturb the overall alignment.

### Self-Consistency

A HMM profile was created using the HMMER program for all the MoLFunCs (including the authority protein) and used as query to search the genomes of species corresponding to the MoLFunCs. A threshold of 10 bits was allowed to check if the original MoLFunC within each species (that is already in the profile) could be found as the best hit in the search results for that species.

### Tools used

BLAST version 2.2.15 was used for the MoLFunC identification process. The BLAST searches were carried out at the level of proteins using the *blastp* option. HMMER version 2.3.2 was used for the refinement and the consistency checks. Both tools are widely used for detecting homologies. In order to determine which multiple sequence alignment algorithm to use, we did a BLAST search of the non-redundant database using Kv1.2 as probe. We picked the least significant hit that had very low percent identity with the probe and aligned it with the probe using CLUSTALW [24] and MUSCLE [19]. MUSCLE outperformed CLUSTALW in aligning the voltage sensor and the selectivity filter. Since these are critical regions for determining functional equivalence, we adopted MUSCLE to align our sequences. The CLUSTALW version used was 1.83 and the MUSCLE version was 3.6. Phase I of the MoLFunC identification process was implemented using Perl scripts. The refinement phase was carried out using HMMER and Excel spreadsheets. Spreadsheets S1, S2 (Supporting information) show the output of our algorithm as applied to Kv1.2, at the end of Phase I (the MoLFunC matrix) and each step of Phase II, for both NCBI (Spreadsheet S1) and Ensembl (Spreadsheet S2) searches.

## Results

Tables 3–6 summarize the number of MoLFunCs obtained using our algorithm and number of orthologs obtained from OMA for each protein. Table 3 shows the comparison across all species that were used as input in either technique. Table 4 is the subset of Table 3 that passed the topology comparison test (described in Methods). Table 5 shows comparison between MoLFunCs and OMA for species that are common (in the input set) to both methods. Table 6 is a subset of Table 5 based on the topology comparison test. In order to achieve a high degree of confidence in functional equivalency within our dataset, we carried out a series of tests, the results of which are outlined below.

**Table 3:** Count of MoLFunCs/orthologs.

	Number of Functional Equivalents in species that are present in results of both methods			Number of Functional Equivalents in species that are present in results of one technique only		
	MoLFunC	UniqueOMA	MultipleOMA	MoLFunC	UniqueOMA	MultipleOMA
HCN1	19	19	0	5	6	0
Gamma6	14	13	1	6	1	0
Kv1.2	21	17	4	9	5	2
Glur	24	23	1	3	10	4
Cng1	18	18	0	1	16	0
Kir7.1	27	26	1	1	15	1
Trpv1	17	17	0	4	12	3

This includes all species that were in input of each method but not necessarily in both. Therefore the numbers of hits that are found in one but not the other do not necessarily represent disagreement. The results represent counts before topology verification.

**Table 4 pone-0005898-t004:** Count of MoLFunCs/orthologs.

	Number of Functional Equivalents in species that are present in results of both methods			Number of Functional Equivalents in species that are present in results of one technique only		
	MoLFunC	UniqueOMA	MultipleOMA	MoLFunC	UniqueOMA	MultipleOMA
HCN1	16	15	0	1	3	0
Gamma6	12	10	1	0	0	0
Kv1.2	22	16	4	9	5	2
Glur	21	19	1	3	8	4
Cng1	16	17	0	1	9	0
Kir7.1	25	25	1	0	8	1
Trpv1	8	7	0	1	3	0

This includes all species that were in input of each method but not necessarily in both. The results represent counts AFTER topology verification of the results in Table 3.

**Table 5 pone-0005898-t005:** Count of MoLFunCs/orthologs.

	Number of Functional Equivalents in species that are present in results of both methods			Number of Functional Equivalents in species that are present in results of one technique only		
	Mf	UniqueOMA	MultipleOMA	Mf	UniqueOMA	MultipleOMA
HCN1	19	19	0	4	4	0
Gamma6	14	13	1	6	0	0
Kv1.2	21	17	4	6	1	0
Glur	24	23	1	3	4	0
Cng1	18	18	0	0	10	0
Kir7.1	27	26	1	1	1	0
Trpv1	17	17	0	4	8	1

This includes all species that were in input of BOTH methods. The results represent counts BEFORE topology verification.

**Table 6:** Count of MoLFunCs/orthologs.

	Number of Functional Equivalents in species that are present in results of both methods			Number of Functional Equivalents in species that are present in results of one technique only		
	Mf	UniqueOMA	MultipleOMA	Mf	UniqueOMA	MultipleOMA
HCN1	16	15	0	1	1	0
Gamma6	12	10	1	0	0	0
Kv1.2	21	16	4	6	1	0
Glur	21	19	1	3	4	0
Cng1	16	17	0	0	4	0
Kir7.1	25	25	1	0	1	0
Trpv1	8	7	0	1	1	0

This includes all species that were in input of BOTH methods. The results represent counts AFTER topology verification. Comparison of Table 5 and 6 shows that most differences between OMA and MoLFunCs are removed by verification that the topology of the hit is the same as the topology of the authority.

### Topological Congruence

**Figure 4:**
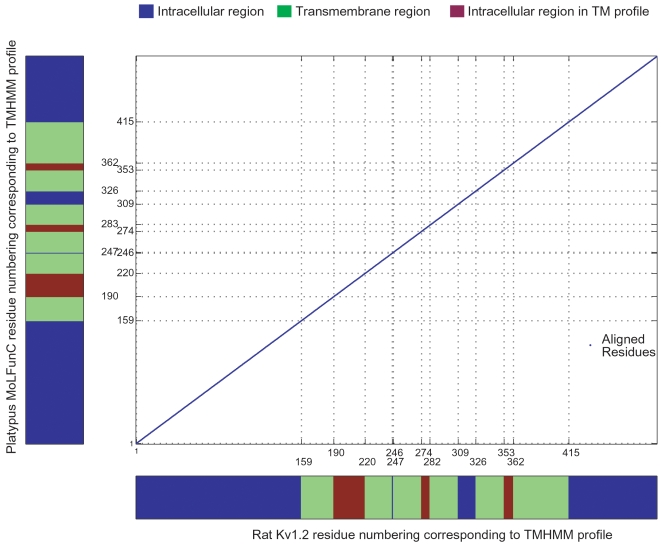
Topological comparison matrix of Kv1.2 (X-axis) versus the MoLFunC from platypus (Y-axis). The central plot gives the alignment dotplot, the grid lines on this plot are the boundaries of TMs. The colored bars near the X and Y-axes are TM profiles derived from TMHMM [2]. Blue indicates intracellular, green – TM regions and red – extracellular regions.

Structural similarity of the MoLFunC set with the authority protein would be a strong indicator of functional equivalence. However, since structure prediction is a difficult task, we rely on congruence of TM topology, as determined by TMHMM. We establish congruence visually as shown in Figure 4. The blue region indicates intracellular side, the green indicates TM regions and the red indicates extracellular side. The plot is the Dot plot of similarities between Kv1.2 and one of the MoLFunCs (platypus). Almost all the MoLFunCs show good topological congruence with Kv1.2. For voltage-gated channels, some of the proteins had the S4 missing based on TMHMM in spite of our thresholds, but aligned relatively well in that region; so we included them in our analysis. Spreadsheets S3, S4, S5, S6, S7, S8 and S9 (Supporting information) have a column showing the results of the topology filtering for both the MoLFunCs and the OMA results. For example, the honeybee MoLFunC from our methodology for Kv1.2 had all TMs, while the honeybee ortholog that was predicted by the OMA browser is missing the S6 segment and hence may not be able to replicate the full functionality of the channel.

### Self-Consistency

We checked for self-consistency of the MoLFunC set, based on the following premise: A HMMER profile of all the MoLFunCs, when used as a probe against each of the genomes, should pick up as best hit the representative MoLFunC that is already in the profile. Spreadsheets S3, S4, S5, S6, S7, S8 and S9 (Supporting information) have a column describing the results for the self-consistency tests for the MoLFunC datasets. On an average, we conclude that the MoLFunC set is 93% self-consistent. The 93% figure puts a number on the phrase “Most likely” in MoLFunCs; i.e., we conclude that each MoLFunC identified is over 90% likely to be the correct one, relative to rest of the set. We did not test the OMA results for self-consistency, because we do not have detailed access to the complete OMA algorithm.

### Conserved Motif Analysis

We examine the conservation patterns of residues/motifs for each MoLFunC set, based on experimental evidence of their importance for specific functional characteristics that all proteins in that set should display. Note that this is a test satisfying necessity constraint, but not sufficiency, since it is limited by the amount of experimental evidence. Therefore, we have not included this in the histogram analysis. The analysis represents one more line of evidence to help ascertain the level of confidence we can place in the functional equivalency of our dataset. Spreadsheets S3, S4, S5, S6, S7, S8 and S9 (Supporting information) have columns detailing the results of the conserved motif analysis. A description of the color codes and columns for these spreadsheets is provided in the document – SuppInfoDescription S1 (Supporting information). Following are the motifs that were considered for each protein:

#### Kv1.2 (Spreadsheet S3)

The voltage sensor in S4 for Kv1.2 has a typical (R/K) pattern every third position. This pattern repeats 7 times. The actual number of repeats could be important; the positively charged residues are thought to be important for the channel's response to voltage changes; the nature of response might depend on the number of these repeats, as by site-directed mutagenesis experiments [25]. Therefore to be consistent with Kv1.2's voltage sensing properties, we imposed the constraint of having the same pattern 7 times in all the MoLFunCs. The only protein that did not satisfy this constraint was from *C.elegans*, which had 6 repeats. The selectivity filter motif *TTVGYG* is also central to the channel function. This motif is conserved in all the species. The last motif that is relevant to gating is *G(10x)G(6x)PVP* in the S6 inner helix [13], [26] , where *x* denotes any residue. This motif is conserved in all species except the *sea urchin*, which has an Alanine instead of the first Glycine. However, on closer inspection, we note that there is a Glycine immediately preceding it (an added feature of many of the proteins in this set). This could be a case of reciprocal mutation and thus we can assume that the sequence is at least partially correct. Overall, the motif conservation constraint is a validation step rather than an additional constraint, since all the MoLFunCs determined by the other steps displayed all expected motifs.

#### Trpv1 (Spreadsheet S4)

Trpv1 channels are sensitive to a variety of stimuli. One of the residues critical for capsaicin sensitivity is S512 [27]. We see that this residue is conserved in all but 3 of the MoLFunCs. D647 is important for preserving pore properties and mutations in this region affect permeation of Ca2+ and Mg2+ [28]. This residue is completely conserved in all except one in which it is missing.

#### CNG1 (Spreadsheet S5)

These channels are nonselective cation channels that are gated by cAMP or cGMP. Residue E365 in human CNG1 (corresponding to E363 in bovine) was identified as a critical binding site for Ca2+ ions that can reduce selectivity for monovalent cations in a voltage dependent fashion [29]–[31]. This residue is completely conserved among the MoLFunCs. Residue N329 in human (corresponding to N327 in bovine) was identified [32] as a N-glycosylation site that can affect channel's electrostatic profile. This residue is also completely conserved among all the MoLFunCs.

#### HCN1 (Spreadsheet S6)

HCN1 channels that contribute to the hyperpolarization activated pacemaker current are sensitive to external Cl ions, the characteristics of which depend on residue A352 [33]. This is conserved in all except 3 species. Residue A354 which was shown to be very important for gating is also conserved in all but 2 species [34]. The selectivity filter signature sequence CIGYG [35] is conserved in all but 3 species. R286 at the end of the S4–S5 linker [36] is important for regulation by voltage and this is conserved in all but one species.

#### Glur (Spreadsheet S7)

Glutamate receptor channels are thought to have structural similarities with K+ channels. Studies aimed at identifying motifs relevant to specific channel properties [37] identified the profile: *[WFLVIM]-[WFY]-[WFYL]-X(6)-[STNQ]-X-G-X(1,3)-[ED]-X(1,2)-P*. This profile is matched in all but one of the MoLFunCs. Another commonly conserved motif *SYTAANLAAF* in glutamate receptors [38], [39] is conserved in all but one of the MoLFunCs.

#### Gamma6 (Spreadsheet S8)

It was recently reported the TM1 of gamma6 was critical to its regulation of Cav3.1 channels. A motif GXXXA was identified as important in this region [40]. The G42 and A46 were important in terms of their being small in size as mutations that increased the size of residues at these positions interfered with current regulation. In our alignment, we see that at position 42, there are mostly small residues including G,A and V. 5 of the species are missing this motif completely. In place of A49, some of these have serine, which is allowable, and one has a threonine, which is chemically similar to serine.

#### Kir7.1 (Spreadsheet S9)

Kir7.1 belongs to a class of inward rectifying channels that are pH sensitive. H26 has been shown to be critical for this sensitivity [41]. In our MoLFunC set, all but 2 of the MoLFunCs have this residue conserved. The same trend can be seen for the rest of the residues/motifs. These include the selectivity filter signature [42] – G(F/Y)G, 3 mutations that were important for K+ conductance [42] - L118, T116 and F156, and a residue that is characteristic of Kir 7.1 among the Kir family – M125 [43], [44].

## Discussion

We have demonstrated a technique based on authoritatively annotated sequences and BLAST score relaxation to extract MoLFunCs (Most Likely Functional Counterparts) from a set of species. We pooled the NCBI and Ensembl databases in order to extract the largest possible sequence set for analysis. Based on our internal consistency checks, we believe that our identification of MoLFunCs is over 90% accurate. The inclusion of an error margin in BLAST score rankings suggests that the set of MoLFunCs will be relatively stable to isolated changes in the databases due to updates, and to BLAST program versions or choices of substitution matrices that might shuffle the top hits based on their scores/e-values.

Since our method is based on similarity of protein sequences rather than inference of patterns of gene descent, it does not explicitly address the issue of in-paralogs and out-paralogs that have been explored by several ortholog prediction approaches [6], [10] and filtering algorithms [45]. However, it seems likely that MoLFunCs would be coded for by orthologous genes. This is due to the refinement step, which implicitly incorporates filtering to arrive at unique representatives. From a comparison with the OMA results, we find that in some cases our MoLFunCs are not found by OMA, and in other cases we find a 1-1 correspondence where OMA finds 1-many. On the other hand, we find some cases where OMA has uncovered a correspondence that MoLFunCs has not, and the OMA correlate passes through our filters. Therefore the two techniques can complement each other to find the most complete sets of corresponding proteins. An exhaustive comparison is presently beyond the scope of this study.

Our technique attempts to integrate knowledge the user brings to the problem with methods of statistical inference. This strategy is independent of the use of any specific tools such as BLAST and HMMER and can incorporate more stringent or permissive algorithms if needed. We believe that this strategy is amenable to newer definitions of authority. In the examples in this paper authority is identified by our knowledge of experiments that definitively characterized the topology and function of the prototypical sequence in each class. One could imagine authority identification using automated information mining from various resources related to the proteins of interest. A concept-based strategy (the concept in this case being that of authority) provides an ontological scaffold to analyze the global network of protein homologies. We envisage that a similar strategy could be applied to analysis of protein interaction networks as well. Although we have applied user knowledge only at the initial stage in our strategy, we can refactor the code to allow for user input at different checkpoints within the algorithm. As a prelude to this goal, our software computes Phase I (preliminary identification) and Phase II (refinement) separately. Therefore, a MoLFunC matrix that is not yet refined can be enhanced for a new species or recomputed for an existing species with new data, by simply removing the entries for that species from the rows and columns of the MoLFunC matrix and restarting Phase I. Our code is designed to take as input an existing MoLFunC matrix and enhance it with new MoLFunCs. The MoLFunC matrix thus provides a visually intuitive hook to incorporate user input and could be manipulated manually if necessary, for the problem at hand, to reflect the user's judgment about functional equivalence.

The notion of authority in our case is analogous to 100% confidence in its annotation. We also defined our sample set to be the “most likely” functional equivalent. These definitions open the doors for probabilistic modeling of the MoLFunC construction process, wherein the authorities are represented by a certain degree of confidence and the identification of MoLFunCs is essentially a problem in belief propagation, algorithms for which have been explored in the context of other research problems. Our confidence in functional equivalence is partly qualitative; since the reliance on similarity scores makes quantitative considerations implicit adjuncts to our judgment, belief propagation could be used to assign probability values to the degree of functional equivalence. A related issue is the different ontological dimensions that define the “function” of the protein. Functional equivalence could be for a subset of these dimensions and the resulting MoLFunC set should be examined for presence of the relevant dimension. For example, if voltage-sensing apparatus is an aspect of the protein ontology that we are interested in, the MoLFunC set should necessarily contain this aspect. Since motifs and domains map to these dimensions in sequence/structure space, motif/domain based overlap could be used as a check for functional equivalence.

We attempted to retain the consensus agreement strategy used by earlier approaches to comprehensively assign orthologs. The consensus agreement approach could suffer from dissimilarities that arise due to evolutionary divergence of the species pair being investigated. Therefore, a weakly significant score would point to lesser agreements between proteins. Use of PSI-BLAST and/or substitution matrices that take divergence into account, such as implemented in the OMA project using PAM matrices, could help eliminate these effects. As a corollary to this observation, we propose that genome sequencing projects should focus on closing the gaps in phylogenetic space, as it would provide as with a more continuously varying evolutionary landscape to arrive at high-confidence substitution matrices. We limited our analysis to metazoan species; future efforts to find MoLFunCs across all the kingdoms of life would benefit from more sensitive homology detection techniques. One path to do that is to search for similar functional domains, as opposed to searching for similar overall proteins [46].

One situation that can arise in some contexts is the problem of multiple authorities. This can arise when we can authoritatively annotate proteins with the same functional description in more than one species. Thus, multiple authorities give us multiple start points but could also present different MoLFunC matrices in the end. A sound strategy to deal with this issue would be to first BLAST all authorities against each other's genomes. Upon examining the output, either manually or using a machine learning technique, we could derive rules for heuristics such as Bit difference tolerance, relaxation criteria etc. Using these rules we could derive MoLFunCs starting from each of the authorities. We could then extract the common MoLFunCs, create a profile along with the original authorities and search the ambiguous genomes with HMMER profile search.

In an effort to be thorough, we explored incompletely sequenced genomes (and/or those whose proteomes were not complete) in our analysis. If an authority is identified in such a genome, then it is possible that there are very few authority-like proteins in that genome. For example, since voltage gated potassium channels are diverse, if the rat proteome did not contain enough representatives of these channels other than Kv1.2, any protein from another species that is only approximately similar to Kv1.2 and much more similar to some other potassium channel, would still only pick Kv1.2 as the best hit. This could result in spurious MoLFunCs being collected. A solution to this problem is to ensure that the authority genome has sufficient number of sequences and possibly include a significant number of proteins related to the authority (if the authority is part of a large family). In cases when the authority genome is sparse, the best strategy is to find a high-confidence MoLFunC in another genome with sufficient sequence samples and then use that MoLFunC as a pseudo-authority. In our case, the rat genome has sufficient representation in the potassium channel family and could serve as the authority genome.

We hope to extend our strategies to genome-wide studies, especially in the context of inferring interaction networks through evolutionary correlation analysis, and also consider MoLFunCs across a larger set of species that includes microbes and other domains of life. Our current implementation runs on a single processor and it would need days or months to run a larger set of proteins on a larger set of genomes. We are working on developing a parallel implementation of the code to assist in scaling up the analysis.

The scaling up of the analysis to the universe of BLAST results between all genomes can give rise to two issues 1) multiple authorities, that can be dealt with as outlined previously and 2) overlapping hubs. Both problems can arise from emergent properties of the network. The situation here will be very similar to the World Wide Web, where many pages are pointing to more than one authoritative resource. For example, any two websites on a specific topic with large number of inbound links could be considered authoritative on those topics. It is also likely that some of the inbound links to both these authorities arise from the same referring site. By analogy, in the homology network of all proteomes, there is likely to be a set of nodes from different species that are similar in function and have large number of inbound links from proteins in other species. In such cases, even in the absence of authoritative annotations, from a network perspective - these nodes could be considered authorities for that specific function. Overlapping hubs are essentially MoLFunCs picked up by authorities that have different functions. We could use techniques similar to the one we used for resolving NCBI and ENSEMBL conflicts, or develop more stringent criteria based on the authority concept.

In conclusion, we believe MoLFunCs determination can contribute to standardized datasets for comparative genomic analysis. Our method in its current form can be used in conjunction with existing efforts to catalog orthologous groups, especially in cases where functional equivalence is the desired output.

## Supporting Information

SuppInfoDescription S1 Supplementary Information Overview
(0.00 MB TXT)

Spreadsheet S1 (0.02 MB XLS)

Spreadsheet S2 (0.04 MB XLS)

Spreadsheet S3 (0.03 MB XLS)

Spreadsheet S4 (0.02 MB XLS)

Spreadsheet S5 (0.02 MB XLS)

Spreadsheet S6 (0.02 MB XLS)

Spreadsheet S7 (0.03 MB XLS)

Spreadsheet S8 (0.02 MB XLS)

Spreadsheet S9 (0.03 MB XLS)
